# Oxygen Saturation in Closed-Globe Blunt Ocular Trauma

**DOI:** 10.1155/2016/8232468

**Published:** 2016-09-06

**Authors:** Chongde Long, Xin Wen, Liu-xue-ying Zhong, Yongxin Zheng, Qianying Gao

**Affiliations:** ^1^State Key Laboratory of Ophthalmology, Zhongshan Ophthalmic Center, Sun Yat-sen University, 54 South Xianlie Road, Guangzhou 510060, China; ^2^Sun Yat-sen Memorial Hospital, Sun Yat-sen University, 107 Yan Jiang West Road, Guangzhou, China

## Abstract

*Purpose*. To evaluate the oxygen saturation in retinal blood vessels in patients after closed-globe blunt ocular trauma.* Design*. Retrospective observational case series.* Methods*. Retinal oximetry was performed in both eyes of 29 patients with unilateral closed-globe blunt ocular trauma. Arterial oxygen saturation (SaO_2_), venous oxygen saturation (SvO_2_), arteriovenous difference in oxygen saturation (SO_2_), arteriolar diameter, venular diameter, and arteriovenous difference in diameter were measured. Association parameters including age, finger pulse oximetry, systolic pressure, diastolic pressure, and heart rate were analyzed.* Results*. The mean SaO_2_ in traumatic eyes (98.1% ± 6.8%) was not significantly different from SaO_2_ in unaffected ones (95.3% ± 7.2%) (*p* = 0.136). Mean SvO_2_ in traumatic eyes (57.1% ± 10.6%) was significantly lower than in unaffected ones (62.3% ± 8.4%) (*p* = 0.044). The arteriovenous difference in SO_2_ in traumatic eyes (41.0% ± 11.2%) was significantly larger than in unaffected ones (33.0% ± 6.9%) (*p* = 0.002). No significant difference was observed between traumatic eyes and unaffected ones in arteriolar (*p* = 0.249) and venular diameter (*p* = 0.972) as well as arteriovenous difference in diameter (*p* = 0.275).* Conclusions*. Oxygen consumption is increased in eyes after cgBOT, associated with lower SvO_2_ and enlarged arteriovenous difference in SO_2_ but not with changes in diameter of retinal vessels.

## 1. Introduction

Closed-globe blunt ocular trauma (cgBOT) may cause various structural and functional damage in posterior segment, including commotio retinae, traumatic optic neuropathy, and choroid rupture. In most cases, damage was directly impacted on retina or optic nerve. However, cgBOT may also influence the vascular dynamics. For instance, arterial occlusion may occur in cgBOT in rare cases [1]. Disturbance of retinal blood metabolism, which may result in ischemia-reperfusion injury, may also occur due to the elevation of intraocular pressure that is often seen immediately after blunt ocular trauma [2]. Long-term follow-up study showed a significant reduction of ocular blood flow and marked increase of resistance to flow in all retrobulbar vessels in injured eyes compared to unaffected ones. Such ocular blood flow disturbance has no relationship with intraocular pressure [3]. Study also showed that peak systolic velocity of central retinal artery was significantly decreased 4 weeks after injury and this hemodynamic disturbance appeared to correlate with dynamic change of thickness of retinal nerve fiber layer [4]. However, these studies mainly are emphasized on the blood supply of retinal blood vessels, and, as far as we know, no study was reported on SO_2_ and diameter in cgBOT as well as the relationship between SO_2_ and systemic conditions.

Automatic retinal oximetry is a noninvasive device to measure retinal oxygen levels with reliable sensitivity and reproducibility [5]. It is wildly used in research of glaucoma, retinal vein occlusion, diabetic retinopathy, and other fundus diseases [6–9]. The purpose of this study is to evaluate the SO_2_ status and vascular diameter in traumatic eyes and unaffected ones as well as their relationship with systemic parameters such as age, systemic oxygen saturation, blood pressure, and heart rate.

## 2. Patients and Methods

### 2.1. Patients

The study prospectively analyzed the SO_2_ measurements performed in 29 patients with unilateral cgBOT before any treatment. Systolic pressure, diastolic pressure, heart rate, and finger pulse oximetry were also measured. The inclusion criteria were as follows: unilateral blunt ocular trauma of various degree, complete retinal attachment, and transparent refractive media. Exclusion criterion includes history of any previous ocular diseases and any systemic disease which may confound retinal oximetry measurements, severe lens opacity, intraocular hemorrhage such as hyphema, vitreous hemorrhage, and severe retinal hemorrhage which may obstruct the observation of retinal vessels. All patients had been informed and agreed to participate in the study. The study adhered to the tenets of Declaration of Helsinki.

### 2.2. Retinal Oximetry

**Figure 1:**
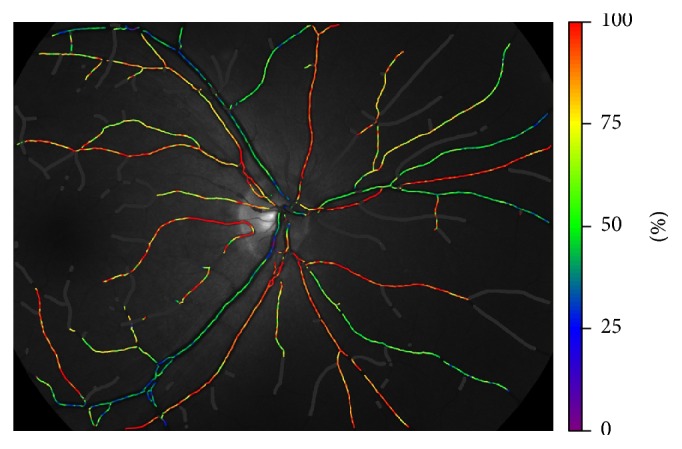
Colors indicate relative oxygen saturation. Scale is on the right side of the image.

The SO_2_ and vascular diameter were measured by retinal oximetry (Oxymap, Inc., Reykjavik, Iceland). The Oxymap system is installed on a fundus camera (Topcon TRC-50DX; Topcon Co., Tokyo, Japan). Retinal oximetry is composed of two cameras including a fundus camera (Canon CR6-45NM; Canon Inc., Tokyo, Japan) which is coupled with a beam splitter (MultiSpec Patho-Imager; Optical Insights, Tucson, Arizona, USA) and a digital camera (SBIG ST-7E; Santa Barbara Instrument Group, Santa Barbara, California, USA). It estimates light absorption of retinal blood vessels at two wavelengths of light, of which one is sensitive to oxygen saturation and the other serves as a reference. It captures fundus images and splits the image into four optic channels. In each of the channels, a filter allows light with the wavelength of 586 and 605 nm to pass. Specialized software automatically selects measurement points on the oximetry images and calculates the optical density (absorbance) of retinal vessels. For such wavelength pairs, hemoglobin SO_2_ is approximately linearly related to the ratio of optical densities [10]. The oximeter is calibrated to yield relative SO_2_ values. Although the calibration is not so perfect that sometimes the measurements exceed 100%, the results are still sensitive and reproducible [5], especially when the results are used for comparison, even if they are different from absolute saturation values. However, the oximeter has been shown to be sensitive to changes in SO_2_. Mean SO_2_ was calculated for first- and second-degree arterioles and venules measuring above 6 pixels in vessel diameter in the measurement zone, which extended from 20 pixels to 220 pixels from the optic disc margin (Figure 1) in both eyes of each patient. Vessels of different segments were matched in traumatic eyes and the unaffected ones. The results were averaged and inserted as one for calculation of the mean for the eye in order to attain this matching. Images were taken in a dark room. The time between images of the same eye was about 1 min. Pupils were dilated with 1% tropicamide (Mydriacyl), which was in some cases supplemented with 10% phenylephrine hydrochloride. The oximeter estimates light absorbance by measuring light intensity outside and inside retinal vessels. Extravascular haemorrhages may therefore interfere with measurements. Care was taken to avoid measuring vessel segments with adjacent haemorrhages to reduce possible errors [9].

### 2.3. Statistical Analysis

Statistical analysis was performed with Statistical Package for the Social Sciences (SPSS) 19. Shapiro-Wilk test was used to test normality of data distribution. Independent sample *t*-test was used to compare SaO_2_ and SvO_2_ value and arteriovenous difference in SO_2_ between traumatic eyes and unaffected eyes. Pearson test was used to evaluate the relationship among SO_2_, diameter of retinal blood vessels in traumatic eyes, duration since trauma, systemic oxygen saturation, systolic pressure, diastolic pressure, and heart rate.

## 3. Results

The mean age of patients at the time of measurement was 36 ± 14 years (range 16–65 years). The mean duration between trauma and measurement was 31 ± 46 days (range 6–180 days). In four patients, the duration was more than three months, and in other patients the duration was less than one month.

### 3.1. SaO_2_ and SvO_2_

**Figure 2:**
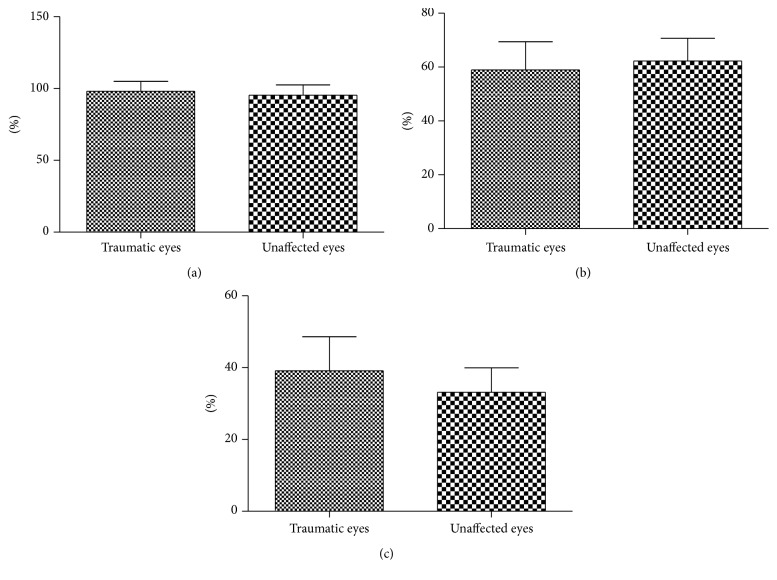
(a) No significant difference was observed in SaO_2_ between traumatic eyes and unaffected ones. (b) SvO_2_ was lower in traumatic eyes than unaffected ones. (c) The arteriovenous difference in SO_2_ was significantly greater in traumatic eyes than in unaffected ones.

Mean SaO_2_ was 98.1% ± 6.8% and SvO_2_ was 57.1% ± 10.6% in traumatic eyes, while the average SaO_2_ and SvO_2_ in unaffected eyes were 95.3% ± 7.2% and 62.3% ± 8.4%, respectively. SvO_2_ was significantly lower in traumatic eyes than unaffected ones (*p* = 0.044), but no significant difference was observed in SaO_2_ (*p* = 0.136). The arteriovenous difference in SO_2_ was 41.0% ± 11.2% in traumatic eyes and 33.0% ± 6.9% in unaffected ones, respectively. The difference value was greater in traumatic eyes than in unaffected ones (*p* = 0.002) (Figure 2).

### 3.2. Arteriolar and Venular Diameter

**Figure 3:**
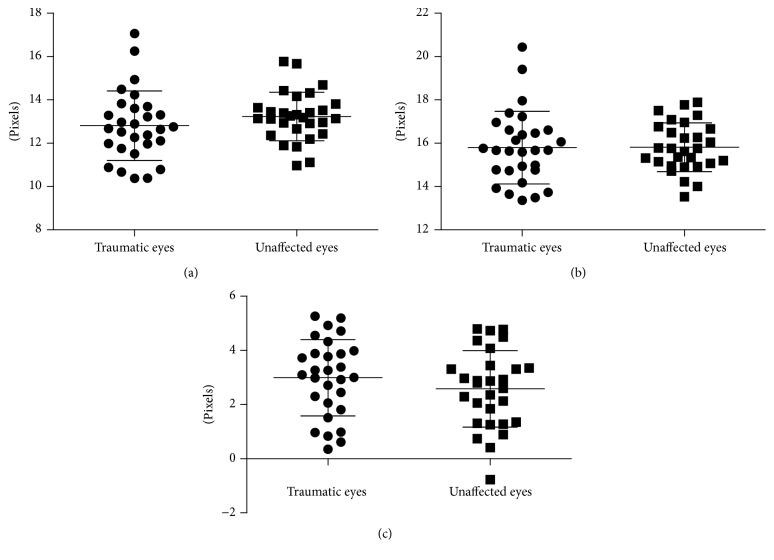
No significant difference was observed in arteriolar diameter (a), venular diameter (b), and arteriovenous difference of diameter (c) between traumatic eyes and unaffected ones.

The arteriolar diameter was 12.8 ± 1.6 pixels and the venular diameter was 15.8 ± 1.7 pixels in traumatic eyes, while the arteriolar diameter and venular diameter in unaffected eyes were 13.2 ± 1.1 pixels and 15.8 ± 1.1 pixels, respectively. The arteriovenous difference in diameter was 3.0 ± 1.4 pixels in traumatic eyes and 2.6 ± 1.3 pixels in unaffected ones. No significant difference was observed between traumatic eyes and unaffected ones in arteriolar diameter (*p* = 0.273) and venular diameter (*p* = 0.916) as well as arteriovenous difference in diameter (*p* = 0.323) (Figure 3).

### 3.3. Other Results

In traumatic eyes, no significant difference was observed between SaO_2_ and arteriolar diameter (*p* = 0.085), duration since trauma (*p* = 0.167), systemic oxygen saturation (*p* = 0.444), age (*p* = 0.666), systolic pressure (*p* = 0.833), diastolic pressure (*p* = 0.246), and heart rate (*p* = 0.509), respectively. There was either no significant difference in traumatic eyes between SvO_2_ and venular diameter (*p* = 0.607), duration since trauma (*p* = 0.250), systemic oxygen saturation (*p* = 0.801), age (*p* = 0.810), systolic pressure (*p* = 0.848), diastolic pressure (*p* = 0.400), and heart rate (*p* = 0.973), respectively. As for arteriovenous difference in SO_2_ of traumatic eyes, there was either no significant difference between it and arteriolar diameter (*p* = 0.055) and venular diameter (*p* = 0.234), duration since trauma (*p* = 0.824), systemic oxygen saturation (*p* = 0.744), age (*p* = 0.526), systolic pressure (*p* = 0.692), diastolic pressure (*p* = 0.965), and heart rate (*p* = 0.564), respectively.

## 4. Discussion

Recent studies concerning cgBOT mainly focus on the pathological, morphological, and functional changes [11, 12], yet few are emphasized on hemodynamic changes in retinal vessels, especially on SO_2_ and diameter of retinal vessels in traumatic eyes. Researches referring to SO_2_ are widely conducted in diabetic retinopathy, retinal vein occlusion, and other diseases [9, 10]. However, no research studies the relationship between cgBOT and SO_2_.

Results in this study show that SaO_2_ is neither higher nor lower in traumatic eyes than in the unaffected ones, while SvO_2_ is significantly lower in traumatic ones. Such condition results in significant enlarged arteriovenous difference in SO_2_ in traumatic eyes. Such phenomenon is attributed to more attraction of oxygen for retinal small arterioles to retinal tissue. The underlying mechanism may be that cgBOT causes retinal or nerve fiber damage, which may lead to increased oxygen consumption of retina, a likely mechanism for repair and clearance of impaired retinal tissue. However, long-lasting damage may lead to irreversible cellular dysfunction [13]. In this stage, the consumption of oxygen may decline, and the enlarged arteriovenous difference in SO_2_ may diminish, as observed in our study that after three months or more, arteriovenous difference in SO_2_ of affected eyes approximates arteriovenous difference of unaffected eyes in four patients. Meanwhile, in two of these patients, the SvO_2_ in traumatic eyes was still significantly lower than unaffected eyes. Also, as Fick's principle stated, the consumption of oxygen is proportional to arteriovenous difference in SO_2_ [14]. So we hypothesize that the arteriovenous difference in SO_2_ is a more sensitive indicator for oxygen consumption.

It is reported that the SO_2_ measurement may be affected by vessel diameter and velocity of blood [15, 16]. However, the impact of vessel diameter appears to be rather small and is unlikely to change the main conclusions [15]. It is consistent with our study, which indicates that the diameter or difference of diameter has no significant difference between traumatic eyes and unaffected ones. Also, in traumatic eyes, the diameter or the arteriovenous difference in diameter has no relationship with the SaO_2_, SvO_2_, and arteriovenous difference in SO_2_, indicating that the oxygen consumption for retinal repair may be not fulfilled by increased blood supply but by more sufficient application of retinal arteriolar SO_2_. The magnitude of the effect of velocity is difficult to estimate as such measurements were not preformed. Oximeter is sensitive to changes of oxygen saturation and produces repeatable results [17], so that technical errors are considered to have little impact on the conclusions of this study.

Some systemic factors in this study seem to have no effect on the measurement of SO_2_. For instance, age was reported to be a factor that decreased retinal SaO_2_ and SvO_2_ [18]. However, it seemed to have no effect on SaO_2_ and SvO_2_ in traumatic eyes in our study. The fact that systemic oxygen saturation had no relationship with SaO_2_ and SvO_2_ was because systemic oxygen saturation is more related to central organs such as lung and heart, and changes in peripheral organs have little effect on systemic oxygen saturation. As for blood pressure, it was reported that history of controlled systemic hypertension was associated with an increase in arteriovenous difference in SO_2_ [18]. But we examine the systolic and diastolic pressure before the measurement of SO_2_ and we found no relationship between them in the traumatic eyes.

Several limitations of our study should be discussed. First, this retrospective study has its intrinsic drawback named as selection bias. Second, the variable follow-up periods and incomplete examination limited definitive conclusions, which may require prospective studies with long and regular follow-up periods, as well as corresponding morphological and functional changes of retinae. Third, several factors may affect the interpretation of results. These factors included incomplete data on supplementary image examination, intraocular pressure and glycemic status, and velocity of blood. Nonetheless, our study is an exploratory study illuminating the oxygen dynamics in cgBOT.

In conclusion, this study demonstrates that SvO_2_ was lower and arteriovenous difference in SO_2_ was larger in cgBOT eyes than in unaffected ones. In cgBOT, oxygen consumption needed for retinal repair may be fulfilled by more sufficient application of retinal arteriolar SO_2_ rather than by increased blood supply. Larger studies will be needed to determine if there is a correlation among SO_2_, visual function, and clinical prognosis in cgBOT.
